# Multi-omics approach reveals gene co-alterations and survival benefit in ovarian cancer patients under platinum-based adjuvant therapy

**DOI:** 10.1016/j.gendis.2025.101628

**Published:** 2025-04-04

**Authors:** Ning Ding, Jiahui Chen, Xiaotian Zhao, Minyi Zhu, Qiuxiang Ou, Jiaohui Pang, Luxi Ruan, Ying Zhang, Wei Sun, Xiaoxiang Chen

**Affiliations:** a Department of Medical Oncology, Wuxi Huishan District People's Hospital, Wuxi, Jiangsu 214187, China; b Department of Gynecologic Oncology, The Affiliated Cancer Hospital of Nanjing Medical University, Jiangsu Cancer Hospital, Jiangsu Institute of Cancer Research, Nanjing, Jiangsu 210009, China; c Geneseeq Research Institute, Nanjing Geneseeq Technology Inc., Nanjing, Jiangsu 210031, China; d Department of Oncology, The Affiliated Jiangning Hospital of Nanjing Medical University, Nanjing, Jiangsu 210009, China; e Department of Pathology, Jinling Hospital, Nanjing University School of Medicine, Nanjing, Jiangsu 210093, China; f Department of Gynecology, The First Affiliated Hospital of Nanjing Medical University, Jiangsu Province Hospital, Nanjing, Jiangsu 210009, China

Key genetic alterations in DNA repair influence the effectiveness of treatments like platinum-based chemotherapy and poly(ADP-ribose) polymerase inhibitors in ovarian cancer, particularly in high-grade serous carcinoma (HGSC). These alterations often include *BRCA1/2* and *TP53* mutations, and their impact is further assessed through homologous recombination deficiency (HRD) derived from genomic instability markers such as loss of heterozygosity and telomeric imbalance.^1^, ^2^, ^3^

This study aimed to identify copy number alteration-based biomarkers alongside HRD scores for ovarian cancer patients receiving platinum-based chemotherapy (Fig. S1). We examined 14 focal copy number alterations in 576 HGSC patients from the TCGA cohort (Table S1) and validated the results in a separate Chinese ovarian cancer cohort (test cohort, *n* = 457). Fourteen pre-identified high-level focal copy number alteration regions, including seven high-level focally amplified and seven high-level focally deleted regions (Table S2), were confirmed by GISTIC in the TCGA cohort (Fig. S2A). HRD scores were calculated using the GeneseeqPrime® HRD pipeline with a cutoff of 43 (Fig. S2B).

The test cohort encompassed HGSC and non-HGSC. Among these, 162 patients had *BRCA1/2* mutations, and 51 had non-*BRCA* HRR-related pathogenic mutations; the remaining 244 had wild-type HRR-related genes. *BRCA1/2* mutations constituted approximately 80% of cases in Chinese ovarian cancer patients harboring at least one HRR-related pathogenic mutation (Fig. S2C), with mutually exclusive occurrences of *BRCA1/2* mutations (*P* < 0.001; Fig. S2D). HRD scores were comparable between *BRCA1*-and *BRCA2*-mutated ovarian cancer but significantly higher than in patients with non-*BRCA* HRR-related pathogenic mutations (*P* < 0.001; Fig. S2E) or wild-type HRR-related genes (*P* < 0.001). Elevated chromosome instability scores were observed in *BRCA1*-mutated samples over non-*BRCA* HRR-related mutations (*P* < 0.001; Fig. S2E), with *BRCA2*-mutated ovarian cancer exhibiting greater chromosome instability scores (*P* < 0.01).

In the TCGA cohort, *BRCA1/2* alteration and *Chr3(q26.2)* amplification (Amp) were positively related with HRD scores, whereas *Chr19(q12)* Amp had a negative impact (Fig. S3A). The association between *Chr3(q26.2)* Amp and increased HRD scores was significant in *BRCA1/2* wild-type (*P* = 0.01; Fig. 1A; Fig. S3B) and *BRCA1/2*-altered HGSCs (*P* = 0.03). *BRCA1/2* wild-type HGSCs with *Chr19(q12)* Amp had significantly lower HRD scores (*P* < 0.01; Fig. 1A). However, *Chr19(q12)* Amp did not significantly impact HRD scores in *BRCA1/2*-altered HGSCs (*P* = 0.49; Fig. S3C), likely due to few *BRCA1/2*-altered HGSCs with *Chr19(q12)* Amp (7.6% *vs.* 25.5%; *P* < 0.001). In the test cohort, a trend towards more prevalent HRD mutational signatures were observed in samples with *Chr3(q26.2)* Amp than those without (43% *vs.* 29%; *P* = 0.08; Fig. 1B). Among samples harboring HRR-related mutations, those with *Chr3(q26.2)* Amp exhibited significantly higher HRD scores than those without, regardless of detectable *BRCA1/2* mutations (*BRCA1/2*: *P* < 0.001; non-*BRCA*: *P* < 0.001; Fig. 1C, D; Fig. S4A, B). Increased chromosome instability scores were observed in those with *Chr3(q26.2)* Amp compared with those without (*P* < 0.01; Fig. 1C, D). Elevated HRD scores (*P* < 0.001; Fig. 1E; Fig. S4C) and chromosome instability scores (*P* < 0.001) were linked to *Chr3(q26.2)* Amp in ovarian cancer with wild-type HRR-related genes.

**Figure 1:**
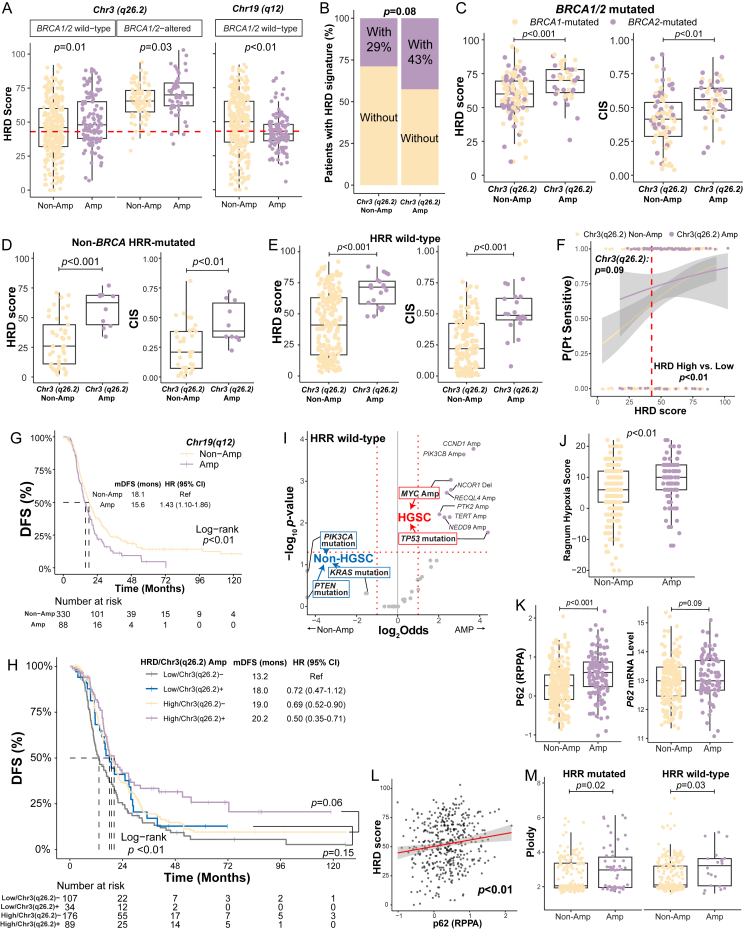
Chromosomal amplifications and homologous recombination deficiency in ovarian cancer. **(A)***Chr3(q26.2)* amplification (Amp) was associated with higher homologous recombination deficiency (HRD) scores in both *BRCA1/2* wild-type and *BRCA1/2*-mutated high-grade serous carcinoma (HGSC) of the TCGA cohort. **(B)** HRD mutational signatures were more common in ovarian cancer with *Chr3(q26.2)* Amp of the test cohort. **(C**–**E)** In the test cohort, *Chr3(q26.2)* Amp was associated with high HRD scores and chromosome instability scores (CIS) in ovarian cancer with pathogenic *BRCA1/2* mutations, with pathogenic mutations of homologous recombination repair (HRR)-related genes other than *BRCA1/2*, and with wild-type HRR-related genes. **(F)***Chr3(q26.2)* Amp and high HRD scores were associated with high probabilities of being sensitive to platinum-based adjuvant therapy. **(G)***Chr19(q12)* Amp was associated with inferior disease-free survival (DFS) in the TCGA cohort. **(H)***Chr3(q26.2)* Amp was able to identify HGSCs benefiting from platinum-based adjuvant therapy. **(I)** The volcano plot of differentially distributed genetic alterations between HRR-related genes in wild-type ovarian cancer with and without *Chr3(q26.2)* Amp. **(J)***Chr3(q26.2)* Amp was associated with high Ragnum hypoxia scores in the TCGA cohort. **(K)***Chr3(q26.2)* Amp was associated with high p62 protein and mRNA expression. **(L)** p62 protein expression was correlated with HRD scores. **(M)***Chr3(q26.2)* Amp was associated with high ploidy in ovarian cancer with and without pathogenic mutations of HRR-related genes.

Among 406 HGSCs in the TCGA cohort receiving platinum-based adjuvant chemotherapy, those with *Chr3(q26.2)* Amp might be more sensitive to platinum-based adjuvant chemotherapy than those without (*P* = 0.09; Fig. 1F), particularly HRD-positive patients (*P* < 0.01). *Chr19(q12)* Amp was linked to a marginally decreased sensitivity possibility (*P* = 0.11; Fig. S5A) and poorer disease-free survival (*P* < 0.01; Fig. 1G). HRD-positive HGSCs with *Chr19(q12)* Amp showed relatively poor disease-free survival compared with those without (*P* = 0.06; Fig. S5B). However, we observed a trend towards better disease-free survival in HRD-positive patients with *Chr3(q26.2) Amp* (*P* = 0.06; Fig. 1H). A multivariable Cox regression model confirmed *Chr3(q26.2)* Amp's strong association with superior disease-free survival (*P* < 0.01; Fig. S5C). Our test cohort showed a similar trend (*P* = 0.15; Fig. S5D), although it did not achieve statistical significance. HRD-positive patients demonstrated a trend toward better disease-free survival (*P* = 0.18; Fig. S5E). The difference in HRD-negative patients was not obvious (*P* = 0.47), possibly due to the small sample size. In the TCGA cohort, HGSCs with *Chr3(q26.2)* Amp had a marginally better overall survival than those without (*P* = 0.09; Fig. S6A), with a similar trend observed in the multivariable Cox regression model (*P* = 0.12; Fig. S6B).

In the test cohort, *Chr3(q26.2)* Amp was enriched in ovarian cancer with *BRCA1/2* mutations compared with those without (*P* < 0.001; Fig. S6C). This prevalence was similar in the TCGA cohort for *BRCA1/2*-mutated samples (*P* = 0.14; Fig. S6D) and higher in HGSCs without *BRCA1/2* mutations (*P* < 0.001). Given that *Chr3(q26.2)* Amp could occur in ovarian cancer with wild-type HRR-related genes, we performed the comparison of concomitant genetic alterations in HRR wild-type samples between those with and without *Chr3(q26.2)* Amp in the test cohort (Fig. 1I). *TP53* mutations and amplified genes like *MYC* and *CCND1* were enriched in samples harboring amplified *Chr3(q26.2)*. *PIK3CA* and *PTEN* mutations were only identified in samples without *Chr3(q26.2)* Amp. 94 % of samples without *Chr3(q26.2)* Amp had *PIK3CA* mutations, whereas *PIK3CA* Amp were predominantly carried by ovarian cancer harboring *Chr3(q26.2)* Amp (*P* < 0.001; Fig. S6E). Most *PIK3CA*, *PTEN*, and *KRAS* mutations in samples without *Chr3(q26.2)* Amp were annotated as oncogenic/likely oncogenic by OncoKB™, such as *PIK3CA*^*H1047R/Y*^, *PTEN*^*G132D/V*^, and *KRAS*^*G12D/V/S*^ (Fig. S6F). These differently enriched ovarian cancer driver mutations demonstrated that *Chr3(q26.2)* Amp was enriched in HGSCs harboring *TP53* mutations and *MYC* Amp; however, non-HGSCs driven by *PIK3CA*, *PTEN*, or *KRAS* mutations were less likely to carry *Chr3(q26.2)* Amp.

HGSCs with *Chr3(q26.2)* Amp had significantly higher Ragnum hypoxia scores (*P* < 0.01; *Q* = 0.04; Fig. 1J). Interestingly, the KEGG analysis based on RNA sequencing data revealed up-regulated immune-related pathways in samples with *Chr3(q26.2)* Amp, while those without exhibited up-regulated Hedgehog signaling pathway (Fig. S7A). Four proteins were differentially expressed, including PIK3CA, p62 (SQSTM1), CCNB1, and TSC1 (*Q* < 0.05; Fig. S7B). Elevated p62 protein expression and mRNA levels were noted in *Chr3(q26.2)* Amp HGSCs (*P* < 0.001 for protein; *P* = 0.09 for mRNA; Fig. 1K), correlating positively with HRD scores (*P* < 0.01; Fig. 1L). PIK3CA protein and mRNA levels were significantly higher in *Chr3(q26.2)* Amp patients (*P* < 0.001; Fig. S7C), with 87.2% HGSCs with amplified *PIK3CA* also showing *Chr3(q26.2)* Amp (Fig. S7D). CCNB1 expression was higher in HGSCs with *Chr3(q26.2)* Amp than those without, with a similar trend observed in mRNA (*P* = 0.03; Fig. S7E). Increases in CDK1 protein (*P* = 0.03; Fig. S7F) and mRNA of GMNN (*P* < 0.01) were also noted in these samples. Additionally, increased ploidy associated with *Chr3(q26.2) Amp* was observed in our test cohort, affecting samples with (*P* = 0.02; Fig. 1M) and without HRR-related mutations (*P* = 0.03).

We hypothesize that *Chr3(q26.2)* Amp flags hypoxic ovarian cancer tumors with genomic instability, p62 accumulation from *TP53* mutation-induced autophagy deficiency, and suppressed HRR pathway, leading to persistent DNA damage and cell cycle arrest (Fig. S7G). Our findings demonstrated that *Chr3(q26.2)* Amp was associated with elevated HRD scores, chromosome instability, and enhanced platinum-based adjuvant chemotherapy outcomes, particularly in HGSCs with a hypoxic and impaired HRR environment. *Chr3(q26.2)* and *Chr19(q12)* Amp significantly impact HRD and chemotherapy efficacy. Consistently, Wang et al found that *MECOM* amplification on *Chr3(q26.2)* was correlated with a favorable prognosis through HRD mutational signature and structural rearrangement.^4^ Additionally, p62's interaction with RNF168 impedes the recruitment of key DNA repair proteins such as BRCA1 and RAD51,^5^ suggesting that p62 accumulation might reduce HRR efficiency, thereby enhancing platinum-based adjuvant chemotherapy response in *Chr3(q26.2)* Amp HGSCs.

Overall, *Chr3(q26.2)* Amp could assist in identifying patients with favourable clinical outcomes under platinum-based adjuvant chemotherapy. Consistent results between the TCGA and test datasets revealed the robustness and generalizability of our findings, while further studies are required to confirm the value of *Chr3(q26.2)* Amp in HRD-low patients and the mechanisms behind.

## CRediT authorship contribution statement

**Ning Ding:** Writing – review & editing, Writing – original draft, Formal analysis. **Jiahui Chen:** Writing – review & editing, Writing – original draft, Formal analysis. **Xiaotian Zhao:** Writing – review & editing, Writing – original draft, Visualization, Formal analysis, Data curation. **Minyi Zhu:** Writing – review & editing, Visualization, Formal analysis, Data curation. **Qiuxiang Ou:** Writing – review & editing, Writing – original draft, Visualization, Supervision, Formal analysis, Data curation. **Jiaohui Pang:** Writing – review & editing, Formal analysis, Data curation. **Luxi Ruan:** Writing – review & editing, Funding acquisition, Formal analysis. **Ying Zhang:** Writing – review & editing, Formal analysis. **Wei Sun:** Writing – review & editing, Supervision, Project administration, Conceptualization. **Xiaoxiang Chen:** Writing – review & editing, Supervision, Project administration, Funding acquisition, Conceptualization.

## Ethics declaration

This study was approved by the ethics committee of The Affiliated Cancer Hospital of Nanjing Medical University (NCT05044091). All patients signed informed consent forms before enrollment and sample collection.

## Data availability

The relevant data and its supplemental data can be found in the article or obtained from the corresponding author upon request.

## Funding

This study was supported by the 10.13039/501100001809National Natural Science Foundation of China (No. 81472441 to X. Chen) and the Postgraduate Research & Practice Innovation Program of Jiangsu Province, China (No. SJCX22_0657 to L. Ruan).

## Conflict of interests

X. Zhao, M. Zhu, Q. Ou, and J. Pang are employees of Nanjing Geneseeq Technology Inc., China. The remaining authors have nothing to disclose.
