# Supplementary material for: Multi-omics approach reveals gene co-alterations and survival benefit in ovarian cancer patients under platinum-based adjuvant therapy
Source: Genes Dis. 2025 Apr 4;12(6):101628. doi: 10.1016/j.gendis.2025.101628 (PMC12270775; doi:10.1016/j.gendis.2025.101628)
Supplement: Multimedia component 1 [file mmc1.docx]

**The TCGA cohort**

A total of 576 HGSCs with WES profiles as well as mRNA and protein expression data from The Cancer Genome Atlas Program-OV project were included in the TCGA cohort. The original sequencing and clinical data were downloaded from https://portal.gdc.cancer.gov/projects/TCGA-OV. An in-house developed HRD score pipeline (GeneseeqPrime^®^ HRD, Nanjing Geneseeq Technology Inc., Nanjing, China) was employed to access the HRD status of HGSC samples in the TCGA cohort. Of 576 HGSCs, 406 patients treated with Pt-based adjuvant chemotherapy were included for efficacy and survival analyses (**Fig. S1A**).

**The test cohort**

This study retrospectively included 213 patients diagnosed with ovarian cancer at The Affiliated Cancer Hospital of Nanjing Medical University between June 2020 and May 2023. Patients fulfilling the following inclusion criteria were incorporated in this study: (1) with pathogenic germline and/or somatic mutations of HRR-related genes in tumor tissues; (2) whose tumor tissue biopsies were subject to the HRD evaluation by GeneseeqPrime^®^ HRD (Nanjing Geneseeq Technology, Nanjing, China). Additional 244 HRR wild-type patients with ovarian cancer were also enrolled (**Fig. S1A**). This study was approved by the ethics committee of The Affiliated Cancer Hospital of Nanjing Medical University (NCT05044091) and performed in accordance with the tenets of Declaration of Helsinki. All patients signed informed consent forms prior to enrollment and sample collection.

**DNA extraction, next-generation sequencing, and HRD assessment**

Genomic DNA of tumor tissue biopsies was extracted for sequencing library preparation. Sequencing libraries were captured by probe-based hybridization targeting 437 cancer-relevant genes and over 12000 single nucleotide polymorphisms that evenly distributed throughout the whole genome (GeneseeqPrime^®^ HRD, Nanjing Geneseeq Technology Inc., Nanjing, China). Enriched libraries were sequenced on Illumina HiSeq4000 platform (Illumina, San Diego, CA, USA). HRD scores were calculated using the genome-wide allele-specific copy number estimates including per segment total copy number and minor copy number.

**Pathogenicity identification of *BRCA1/2* and HRR genes**

Somatic or germline *BRCA1/2* mutations were classified as pathogenic mutations if they were nonsense, frameshift, or pathogenic/likely pathogenic in the ClinVar database. Similarly, in addition to *BRCA1/2* mutations, mutations of other 25 susceptible HRR-related genes covered by GeneseeqPrime^®^ HRD panel (**Table S3**) were classified as HRR pathogenic mutations if they were nonsense, frameshift, or mutations defined as pathogenic/likely pathogenic in the ClinVar database.

**Statistical analysis**

Fisher’s exact tests were performed to compare the frequencies of independent subgroups; simple/multiple linear regression models were fitted to test the differences in means. To identify high-level focal CNA regions statistically associated with HRD score, the SHAP value of each focal CNA region was calculated from a fitted XGBoost model. The median follow-up time was estimated using the reverse Kaplan-Meier method. For survival data, Kaplan-Meier curves were generated, and log-rank tests were used to compare differences. Cox proportional hazards models were fitted to estimate hazard ratios (HRs) with 95% confidence intervals (CIs), and the proportionality of hazards was assessed using log(-log) survival plots. Individuals with missing data were excluded from analyses. All quoted *p*-values were two-tailed, and *p*-values <0.05 were considered as statistically significant. Data were analyzed using R software (version 4.2.2), and the *xgboost*, *shapviz*, *survival*, *survminer*, *prodlim*, and *epiR* packages.
