# Supplementary material for: Multi-omics approach reveals gene co-alterations and survival benefit in ovarian cancer patients under platinum-based adjuvant therapy
Source: Genes Dis. 2025 Apr 4;12(6):101628. doi: 10.1016/j.gendis.2025.101628 (PMC12270775; doi:10.1016/j.gendis.2025.101628)
Supplement: Multimedia component 2 [file mmc2.docx]

**Supplementary Figures**


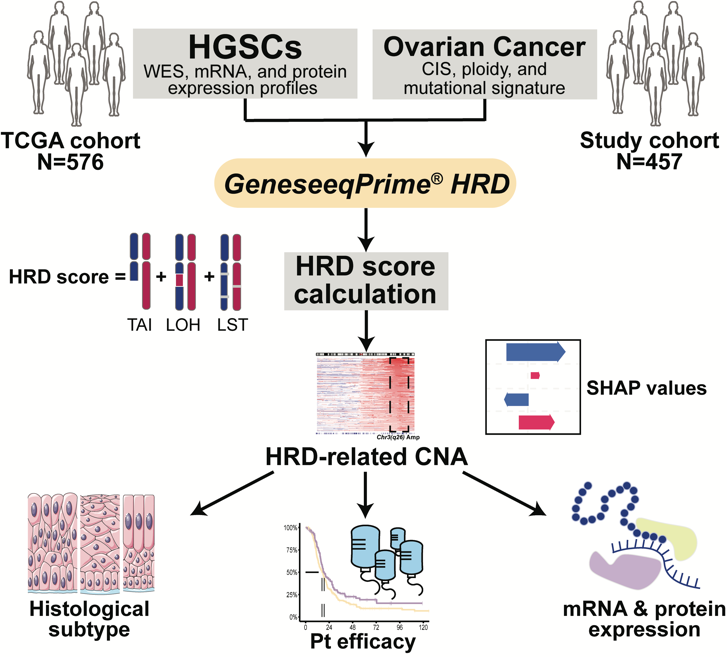


**Figure S1. Patient inclusion and study flowchart.** A total of 576 patients with high-grade serous carcinoma (HGSC) having whole exome and RNA sequencing data were included in the TCGA cohort from the cBioPortal database, of whom 406 patients were treated with platinum-based adjuvant therapy. A total of 213 Chinese patients with ovarian cancer who harbored pathogenic mutations of the homologous recombination repair (HRR) related genes and additional 244 Chinese patients with ovarian cancer who had wild-type HRR-related genes were recruited in the test cohort (N=457), and 78 of them with available prognosis data under platinum-based adjuvant therapy were included for efficacy analyses. The homologous recombination deficiency (HRD) scores were calculated using the pipeline of GeneseeqPrime^®^ HRD.


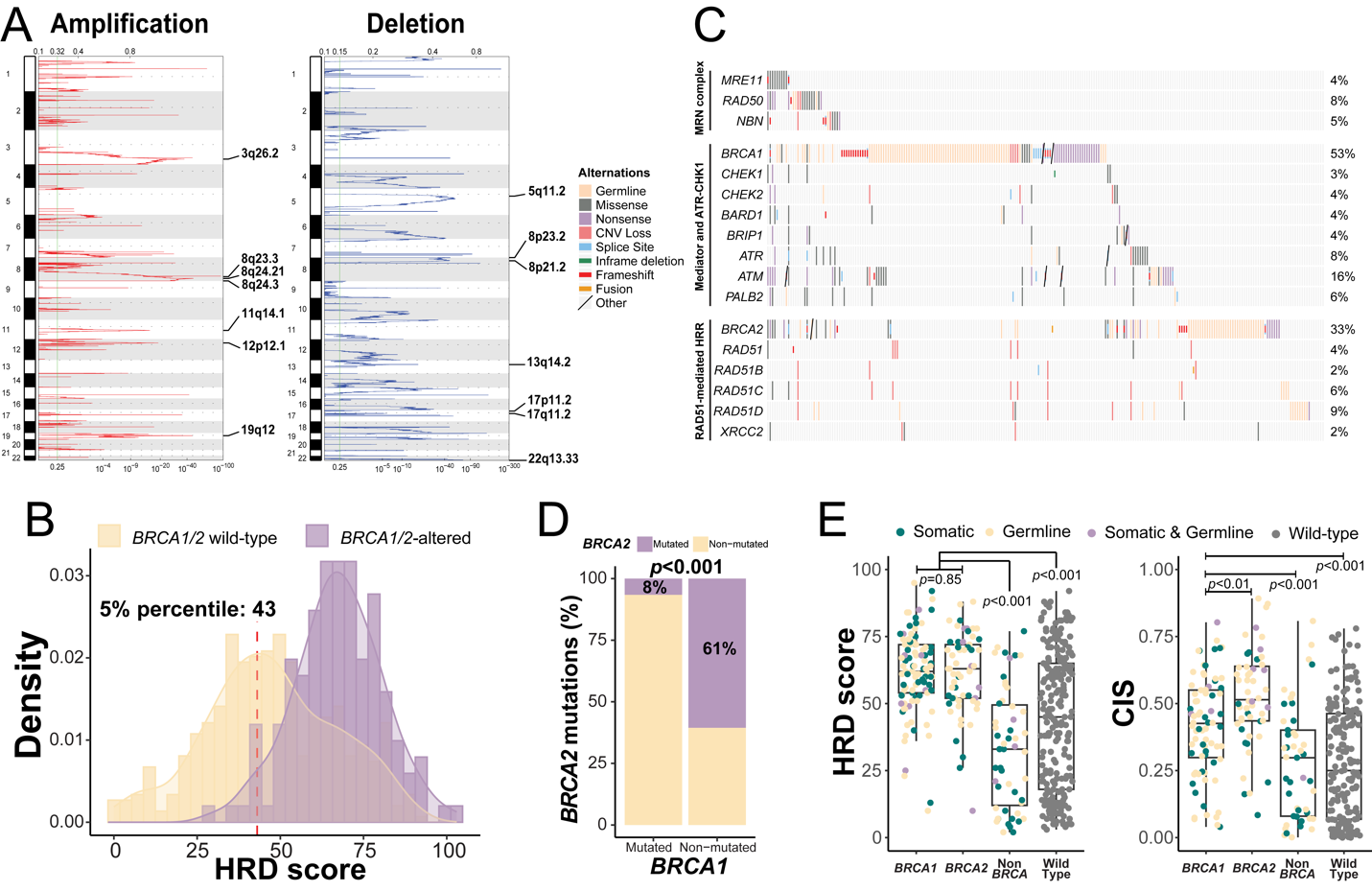


**Figure S2. Genetic alterations and HRD in ovarian cancer.** (A) Seven pre-identified amplified regions were selected, including top five regions with the lowest *q*-values and two regions with low *q*-values and prevalence ≥25%. Seven pre-identified deleted regions with the lowest *q*-values and prevalence ≥5% were also selected. All these 14 high-level CNA regions were double-checked by GISTIC 2.0 in the TCGA cohort. (B) The distribution of HRD scores of the TCGA cohort, with a cut-off of 43 to identify HRD-high and -low patient. (C) The co-mutation plot of homologous recombination repair (HRR)-related genes in 213 patients with ovarian cancer who were identified with at least one pathogenic mutation of HRR-related genes. Most HRR-related genes were classified into three groups, including the MRN complex, the Mediator and ATR-CHK1, and the RAD51-mediated HRR groups. Other few unclassified genes were not displayed here. (D) Mutually exclusive *BRCA1* and *BRCA2* pathogenic mutations observed in the test cohort. (E) Pathogenic *BRCA1/2* mutations associated with higher HRD scores and chromosome instability scores (CIS).


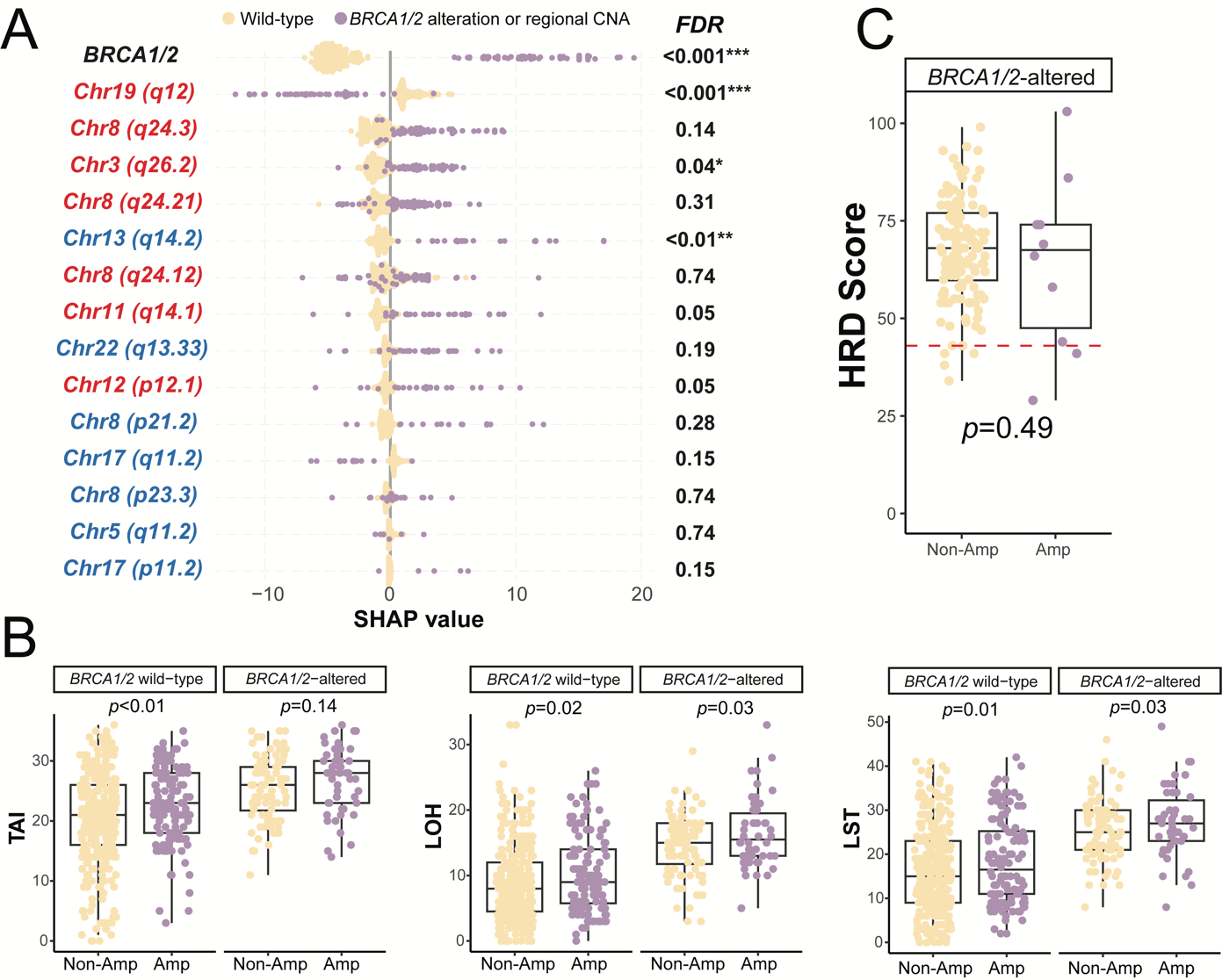


**Figure S3. Influence of CNAs on HRD scores in ovarian cancer.** (A) The SHAP value of each CNA region in a fitted XGBoost model and FDR estimated by a multiple linear regression model. A SHAP value >0 means positive impact on HRD score prediction, whereas a SHAP value <0 means negative influence. The magnitude of SHAP value presents the strength of the influence on HRD score prediction. CNA regions in red: Amps; CNA regions in blue: deletions. (B) Amplified *Chr3(q26.2)* was associated with increased scores of telomeric imbalance (TAI), loss of heterozygosity (LOH), and large-scale transition (LST) in high-grade serous carcinoma of the TCGA cohort. (C) Homologous recombination deficiency (HRD) scores were similar between *BRCA1/2*-altered ovarian cancers with and without *Chr19(q12)* amplification.


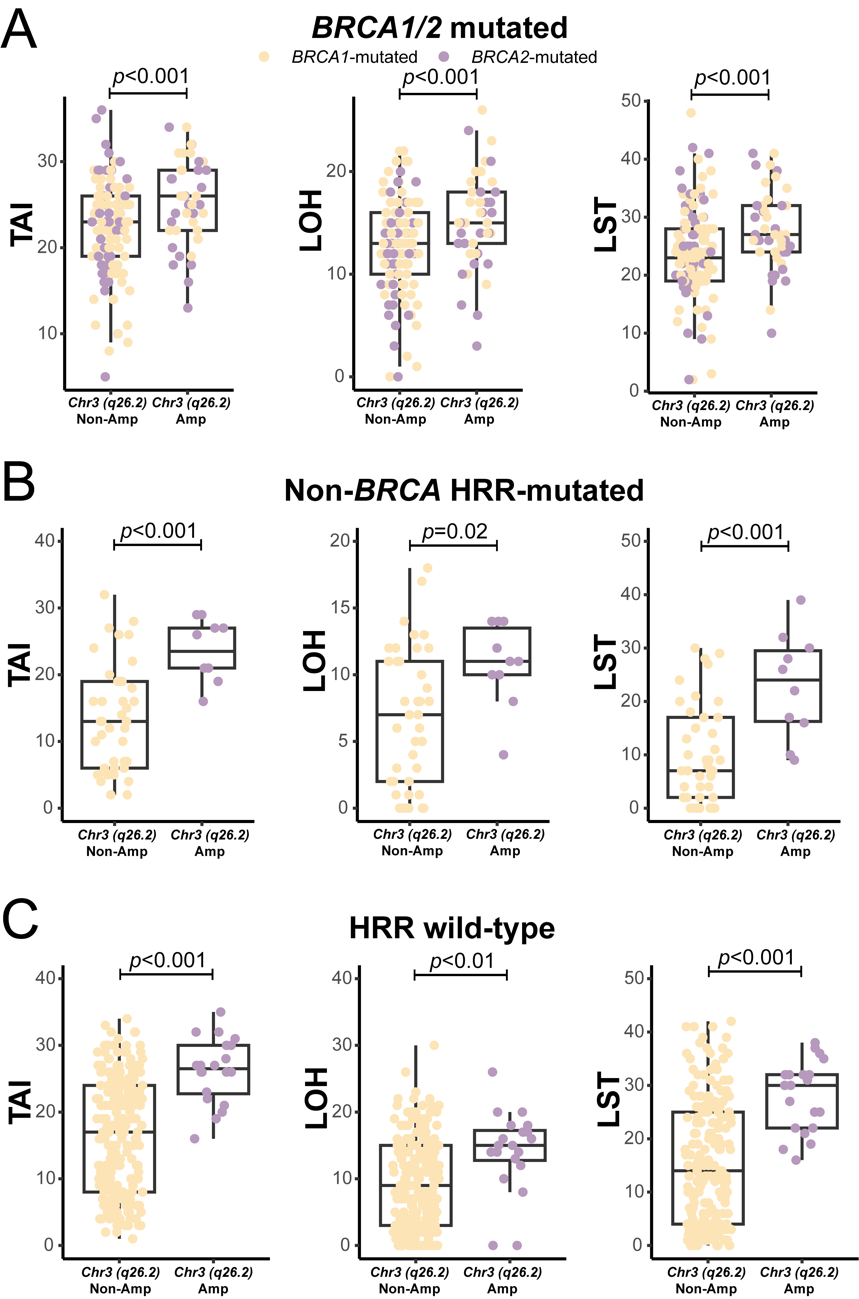


**Figure S4. Impact of *Chr3(q26.2)* amplification** **on** **genomic instability markers in Chinese ovarian cancer patients**. (A) Amplified *Chr3(q26.2)* was associated with increased scores of telomeric imbalance (TAI), loss of heterozygosity (LOH), and large-scale transition (LST) in Chinese ovarian cancer patients with mutated *BRCA1/2*. (B) Amplified *Chr3(q26.2)* was associated with increased scores of TAI, LOH, and LST in Chinese ovarian cancer patients with mutated homologous recombination repair (HRR)-related genes other than *BRCA1/2*. (C) Amplified *Chr3(q26.2)* was associated with increased scores of TAI, LOH, and LST in Chinese ovarian cancer patients with wild-type HRR-related genes.

**
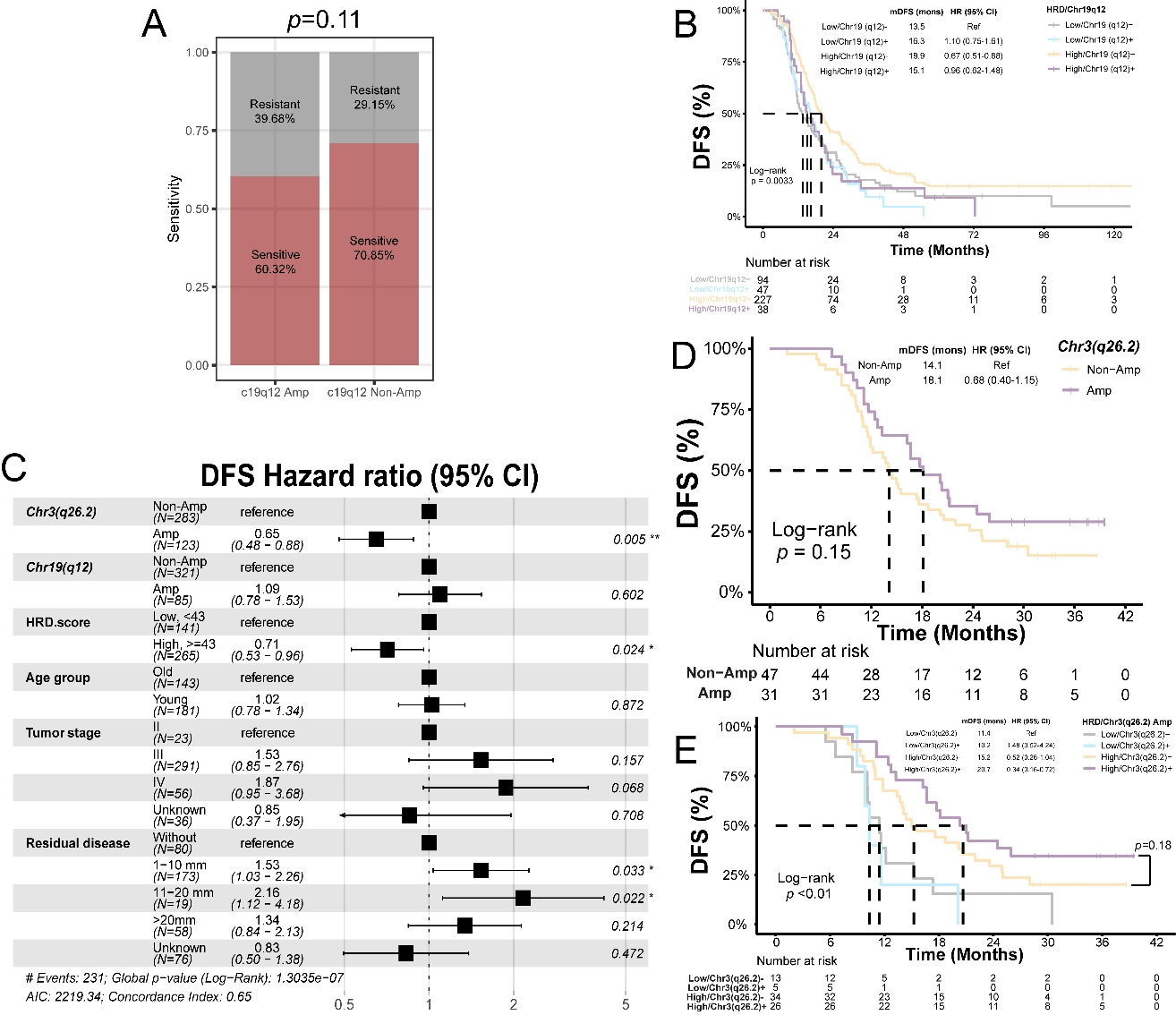
**

**Figure S5. CNA associations and survival outcomes in ovarian cancer.** (A) The sensitivity rates between patients with and without *Chr19(q12)* amplification. (B) The differences in disease-free survival (DFS) among patient groups classified by HRD score and *Chr19(q12)* amplification. (C) In the multivariate Cox regression model, *Chr3(q26.2)* Amp and HRD score were two molecular biomarkers significantly associated with DFS. (D) (E) *Chr3(q26.2)* Amp appeared to be associated with improved DFS under platinum-based adjuvant therapy, especially in patients with a high HRD score.

**
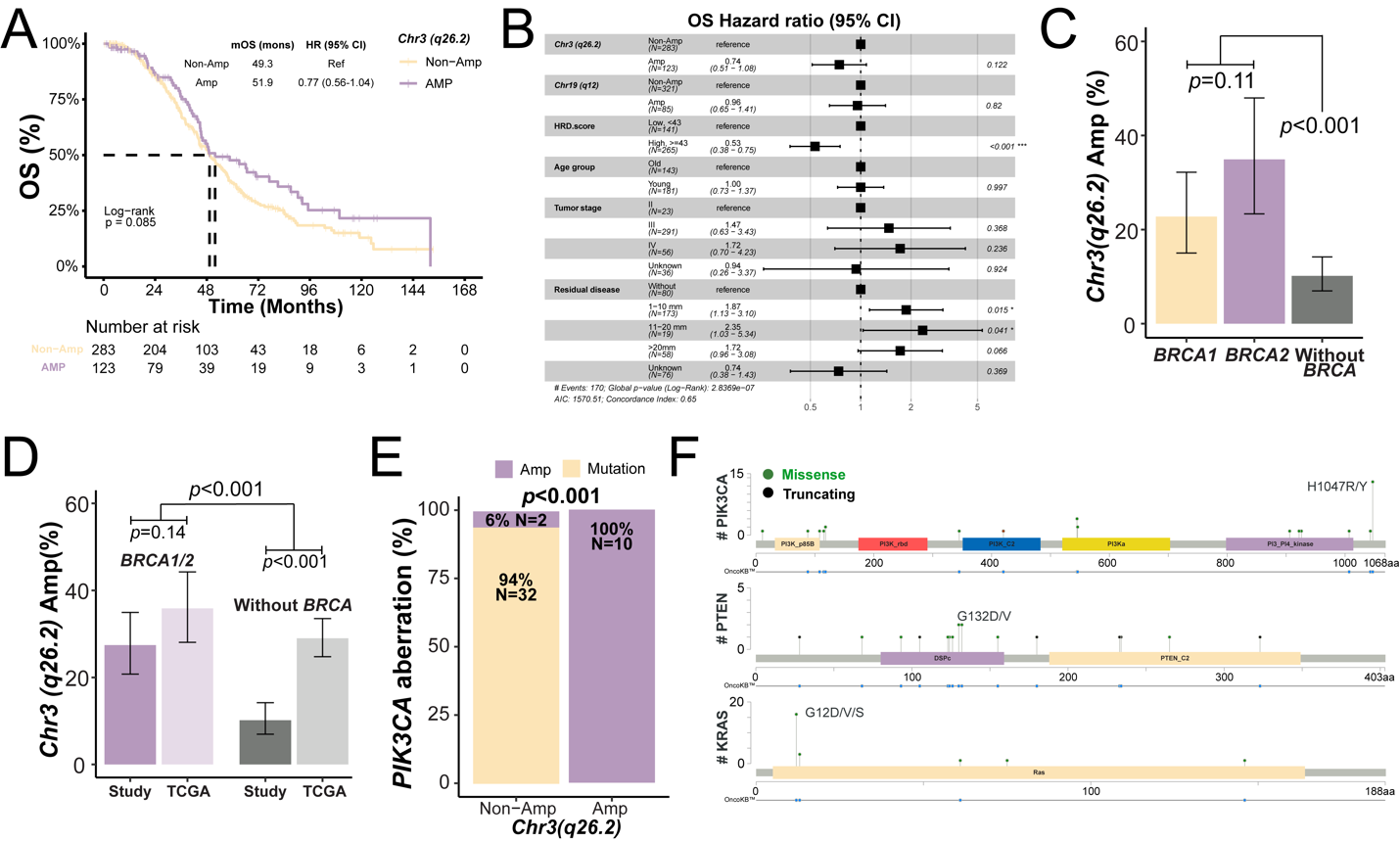
**

**Figure S6. *Chr3(q26.2)* amplification and survival analysis in ovarian cancer.** (A) The overall survival (OS) of patients with and without *Chr3(q26.2)* amplification. (B) The results of multivariable analysis for OS. (C) (D) *Chr3(q26.2)* Amp enriched in ovarian cancer with *BRCA1/2* mutations and more prevalent in the TCGA cohort samples without *BRCA1/2* mutations. (E) Amplified *PIK3CA* and *PIK3CA* mutations and were dominant in samples with and without *Chr3(q26.2)* Amp, respectively. (F) Considerable *PIK3CA*, *PTEN*, and *KRAS* mutations observed in samples without *Chr3(q26.2)* Amp were annotated as oncogenic/likely oncogenic by OncoKB^TM^.


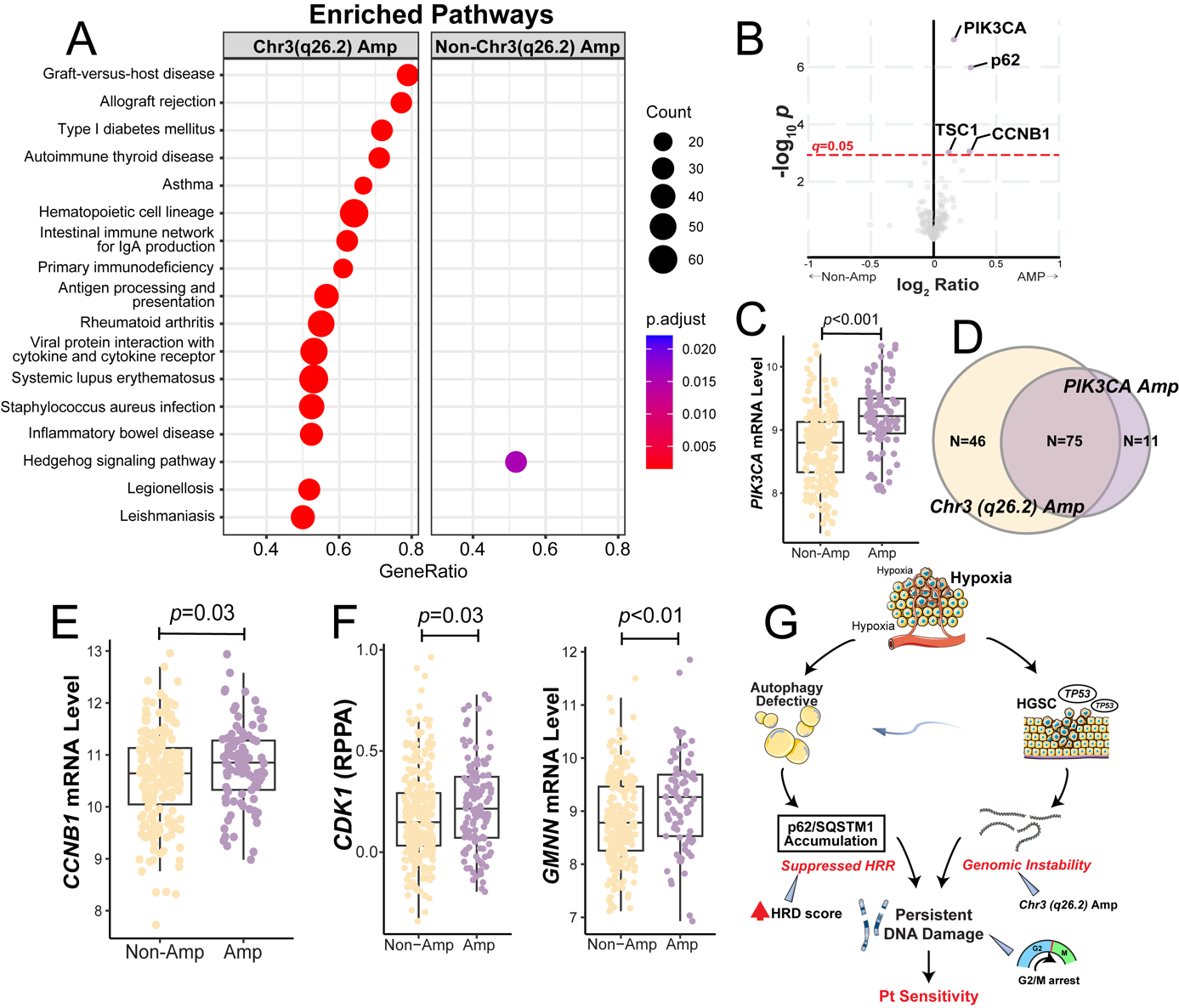


**Figure S7. Clinical and molecular features of ovarian cancer with *Chr3(q26.2)* amplification.** (A) The results of KEGG analysis, with top enriched pathways having gene ratio>0.5 presented. (B) PIK3CA, p62 (also named as SQSTM1), CCNB1, and TSC1 were differently expressed between high-grade serous carcinomas (HGSCs) with and without *Chr3(q26.2)* Amp. (C) Increased mRNA expression of *PIK3CA* in samples with *Chr3(q26.2)* Amp. (D) Amplified *PIK3CA* highly correlated with *Chr3(q26.2)* Amp. (E) Increased mRNA expression of *CCNB1* in samples with *Chr3(q26.2)* Amp. (F) Increased protein expression of CDK1, and increased mRNA expression of *GMNN* in samples with *Chr3(q26.2)* Amp. GMNN protein expression data was unavailable in the TCGA cohort. (G) A supposed model for impaired homologous recombination repair pathway in ovarian cancer with *Chr3(q26.2)* Amp.
