# Supplementary material for: Multi-omics approach reveals gene co-alterations and survival benefit in ovarian cancer patients under platinum-based adjuvant therapy
Source: Genes Dis. 2025 Apr 4;12(6):101628. doi: 10.1016/j.gendis.2025.101628 (PMC12270775; doi:10.1016/j.gendis.2025.101628)
Supplement: Multimedia component 3 [file mmc3.docx]

**Supplementary Table**

**Table S1. Patient characteristics of the TCGA cohort**

| **Characteristics** | **Patients (N=576)** |
| --- | --- |
| Age, median (range), y | 59 (26–89) |
| Histology, No. (%) |  |
| High-grade serous carcinoma (HGSC) | 576 (100.0) |
| Race, No. (%) |  |
| African American | 30 (5.2) |
| American Indian/Alaska Native | 3 (0.5) |
| Asian | 16 (2.8) |
| Caucasian | 402 (69.8) |
| Unknown | 125 (21.7) |
| Disease anatomic site, No. (%) |  |
| Ovary | 477 (82.8) |
| Omentum | 3 (0.5) |
| Unknown | 96 (16.7) |
| Histologic grade, No. (%) |  |
| G1 | 5 (0.9) |
| G2 | 65 (11.3) |
| G3 | 397 (68.9) |
| G4 | 1 (0.2) |
| GB | 2 (0.3) |
| GX | 7 (1.2) |
| Unknown | 99 (17.2) |
| Neoadjuvant therapy, No. (%) |  |
| Yes | 1 (0.2) |
| No | 479 (83.2) |
| Unknown | 96 (16.7) |
| Radiation therapy, No. (%) |  |
| Yes | 4 (0.7) |
| No | 460 (79.9) |
| Unknown | 112 (19.4) |

**Table S2. Fourteen high-level focal copy number alteration regions**

| **No.** | **Chromosome** | **Peak** | **Amp/Del** | ***q*-values** | **Prevalence (%)** |
| --- | --- | --- | --- | --- | --- |
| 1 | chr19 | 19q12 | Amp | 2.5656E-96 | 23 |
| 2 | chr3 | 3q26.2 | Amp | 3.2844E-56 | 27 |
| 3 | chr8 | 8q24.21 | Amp | 3.7003E-56 | 34 |
| 4 | chr11 | 11q14.1 | Amp | 9.2752E-28 | 12 |
| 5 | chr12 | 12p12.1 | Amp | 2.6645E-23 | 11 |
| 6 | chr8 | 8q24.3 | Amp | 4.3368E-20 | 27 |
| 7 | chr8 | 8q24.12 | Amp | 0.000045827 | 26 |
| 8 | chr22 | 22q13.33 | Del | 1.0139E-39 | 9 |
| 9 | chr17 | 17p11.2 | Del | 2.6812E-25 | 10 |
| 10 | chr13 | 13q14.2 | Del | 9.9003E-16 | 8 |
| 11 | chr8 | 8p23.3 | Del | 1.0882E-14 | 9 |
| 12 | chr5 | 5q11.2 | Del | 4.9171E-14 | 5 |
| 13 | chr8 | 8p21.2 | Del | 3.476E-09 | 7 |
| 14 | chr17 | 17q11.2 | Del | 6.578E-08 | 6 |

Amp: amplification, Del: deletion.

**Table S3. 25 covered HRR-related genes**

| *ATM* | *ATR* | *BARD1* | *BLM* | *BRIP1* |
| --- | --- | --- | --- | --- |
| *CDK12* | *CHEK1* | *CHEK2* | *FANCA* | *FANCC* |
| *FANCD2* | *FANCI* | *FANCL* | *MRE11* | *NBN* |
| *PALB2* | *PPP2R2A* | *RAD50* | *RAD51* | *RAD51B* |
| *RAD51C* | *RAD51D* | *RAD54L* | *WRN* | *XRCC2* |
